# Application of Seismic Sensors in Measurement While Drilling

**DOI:** 10.3390/s26030944

**Published:** 2026-02-02

**Authors:** Manoj Khanal, Tianzhu Duan, Yi Duan, Matt Van De Werken, Baotang Shen, Xing Li

**Affiliations:** 1 CSIRO Minerals, Commonwealth Scientific and Industrial Research Organization, 1 Technology Court, Pullenvale, QLD 4069, Australia; 2 CCTEG Chongqing Research Institute, Chongqing 400037, China

**Keywords:** measurement while drilling, accelerometers, geophones, seismic sensors

## Abstract

Rock geotechnical properties can be reflected in drill signals while drill rod penetrates through rocks. The rate of penetration, rotary speed, torque, load, sound, vibration, etc., are different for various rock types, since they are influenced by rock properties. Therefore, a close analysis and derivations of these drill signals can provide valuable insights into rock geotechnical properties. The drill returned signals from the mechanical sensors; for example, torque and load are commonly interpreted to characterize the rock properties. There are still limitations to such sensors and interpretation methodologies that can confidently characterize rock properties. In this research, mechanical sensors were compared and complemented with seismic sensors, for example, accelerometers and geophones, to characterize rocks and interfaces. This paper presents experimental results conducted with synthetic rock samples using mechanical and seismic sensors with a field scale drilling machine. The results show that seismic sensors can identify voids or weak (fractured) interfaces clearly compared to mechanical sensors. Smaller gaps have smaller span of low frequency and vice versa. The sensors attached to the drill head were less sensitive than the sensors attached to the sample. Drill signals showed the capacity to effectively identify material interfaces and weak fractures up to 4 mm thick, with geophones providing clearer data than accelerometers. Neither sensor distinguished fractured zones from voids. Sensors mounted directly on the sample were more sensitive than those attached to the drill head, likely due to vibration-induced signal attenuation at the drill head.

## 1. Introduction

Measurement while drilling (MWD) has been researched over the past decades, along with new sensing techniques, and one of them is using seismic sensors, particularly, geophones and accelerometers, referred to as seismic-MWD or seismic while drilling (SWD). Seismic while drilling (SWD) has been explored by numerous researchers for diverse applications. While it is widely used in the oil and gas sector, its adoption in coal mining is not common. In coal mining, the precise identification of geological strata is essential for designing support systems and ensuring a safe underground environment. Conversely, in the oil and gas industry, geophysical data from seismic logging during drilling are typically employed to estimate the depth and intensity of pressured formations within sedimentary basins.

A recent study by Li et al. [1] found that conventional MWD data are disadvantaged by random fluctuations and poor correlation with geological formations and geo-mechanical properties, and hence undermines the reliability of stratigraphic characterization. Conventional MWD data analysis approach has limitations in predicting rock mechanical properties [2,3]. However, in some cases, the approach seems to fulfill the strata identification purpose [4]. A number of researchers have utilized a range of data analysis techniques, for example, neural network, deep learning, acoustic, cross attention model and wavelet transform [3,5,6,7,8,9,10,11,12], that are complimentary and better than traditional data analysis techniques. The application of the support vector machine approach slightly improved interpretation compared with the traditional MWD approach. In another recent study, the correlation between MWD data and a drillhole investigation was not found to be satisfactory due to the high noise-to-signal ratio [13].

In coal mining, one key application of seismic while drilling is detecting the coal seam top ahead of the drill bit. A study on investigating reverse vertical seismic profiling—by placing geophones on the surface and within a nearby borehole intersecting the coal seam—found that, although the method was relatively simple to implement, its practical use proved challenging and yielded no meaningful results [14]. Similarly, in the oil and gas industry, reviews of seismic while drilling techniques have highlighted that current systems face significant limitations when implementing vertical seismic profiling [15]. Nevertheless, a review of multiple cases in the petroleum industry indicates that reliable seismic signal predictions can significantly enhance drilling design; for example, the implementation of a borehole sensor in the drill tip [16] improves operational safety and reduces costs. However, achieving this requires high-resolution seismic data, advanced computational tools, and more sophisticated analysis techniques [17]. Pilot waves, combined with geophones, can be utilized to capture signals during drill-bit seismic while drilling [18]. The study [19] trailed dual-sensor measurements within drill strings to characterize and isolate drill-bit signal reflections. Based on both synthetic numerical models and real-world measurements, the authors of the paper [19] concluded that positioning dual sensors at an optimal location—ideally as close to the drill bit as possible—can significantly enhance the quality of the SWD pilot signal by eliminating unwanted reflections from the drill string and ensuring accurate reflections from the formation ahead of the bit [19].

In the research [20], it was demonstrated that the real-time transmission of seismic waveforms can reduce uncertainties associated with inverted velocity profiles and enhance risk management during drilling operations. In oil and gas industry, ref. [18] discussed the limitations of both “online” and “offline” data analysis in seismic while drilling. As per the author, the “off-line” approach would quickly fill up internal storage, especially at higher acquisition rates. In contrast, the “on-line” approach allows for only a limited number of signals to be transmitted to the surface in real time, as telemetry systems operate at very low bit rates. The authors proposed a simple algorithm designed for integration with conventional drilling equipment; however, its field implementation has not yet been reported [18].

Rock geotechnical properties can be reflected in drill signals while drill rod penetrates through the rock. The rate of penetration, rotary speed, torque, sound, vibration, etc., are different for various rock types, since they are influenced by rock properties. Therefore, a close analysis and derivations of these drill signals can, at least, provide valuable insights into the rock geotechnical properties. However, at this stage, interpretation and derivations of the drill signals to characterize absolute rock properties is still a challenge. The separation of noise from the actual signal is a challenge due to high noise to signal ratio in the drill signals. A comparative analysis can still be performed from the drilled signals. The interpretation of rock properties for various layers is, nevertheless, valuable in designing and reinforcing support systems. The most commonly used derived parameter to interpret the drilled layer is the specific energy of drilling (SED) [21], which still cannot provide reliability and confidence in interpreting absolute rock properties.

An example of the SED [21] to relate the UCS of samples is demonstrated in demonstrated in [22]. In rotary non-percussive drilling, the work to remove the material was carried out by the thrust and torque [21]. The SED is classified as work carried out per unit volume of the excavated material while drilling. This equation has been modified by various researchers for different modeled conditions (see, [23]) in oil and gas industry.

Finfinger et al. [22] noted that with the thrust component, the fractures and bedding separations can be identified but determination of their dimensions is a challenging task. Rotational speed and penetration rate are found to have greatest effect on the specific energy [22,24]. However, while using specific energy to classify the rock types, a caution should be exercised [25].

In addition to the SED, other interpretation methods, for example, rotational work fraction, rotation-to-thrust power ratio [26], and torque to pressure ratio [27], are also considered by researchers. With the rotation-to-thrust power ratio, ref. [26] were not able to establish a direct relationship between the shear strength of rock strata using MWD data but were able to detect the coal seam without geophysical measurement. In an open-pit copper mine, penetration rate has shown the potential to estimate geological conditions to improve productivity and blast design [28]. However, the author of that study [28] suggested a need for further research. In general, none of the considered methods can be universally applied to interpret rock types using drill data, and still cannot provide absolute properties, such as compressive strength, of the medium being penetrated.

Seismic sensors (i.e., accelerometer and geophone) data are also used to characterize the rock mass properties. Rostami et al. [29] suggested that the vibration signals can accurately detect the joints and rock mass strengths during drilling. Using three-component accelerometers and vibration data, Glubokovskikh et al. [30] inferred rock stiffness with machine learning algorithm based on polynomial regression. Signals received from the accelerometers that are attached to the tunnel boring machine showed that the residual neural network outperformed other tested machine learning methods [31]. Silvestrov [32] used 2500 uniaxial geophones and two triaxial accelerometers, respectively, at the shallow surface and top of the drill to monitor the vibration signal associated with a downhole drilling to 812 m deep. It was found that the seismic signals correlated well with the revolutions per minute (RPM) data collected by the drilling machine. The travel–time curves of pilot signals were compared with the vertical seismic profile, and the results suggested that the travel–time curves reflected the variations of the stratigraphy. Nevertheless, manual time synchronization between data acquisition systems is required to conduct the correlation analysis.

Despite the research progress, the sensitivity and reliability of using seismic signals in detecting the rock mass interface have not been fully investigated, at least in the mining industry. In underground mining applications, cost-effective practices with more robust seismic data analysis methods need to be developed to implement MWD.

One of the challenging tasks encountered in this field of research is the identification of voids, fractures and thin layers present in the strata. To investigate the identification of thinner voids or weak strata as drill bit penetrates the medium, a fundamental experimental setup and approach were adopted in present study. Along with the mechanical sensors, seismic sensors were also instrumented during drilling.

In this research, synthetic rock samples were prepared for the MWD experiment and mechanical and seismic sensors were used. Two different types of seismic sensors were selected for this study: accelerometers and geophones. The signals obtained from the sensors were analyzed to characterize the synthetic rock medium while drill bit penetrated the rock. A widely used machine learning algorithm, Random Forest, was used for classification and regression tasks.

**Figure 1:**
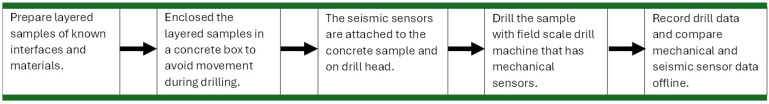
Experimental workflow.

As noted above, despite MWD’s wide application on the oil and gas industry, its application on the coal mining industry is uncommon and has not been reported. Most of the current drill machines used in the mining industry have mechanical sensors and acquire data, but these data are not used for any “on-line” or “off-line” interpretation of the drilled medium. This could be due to the lack of confidence in current data acquisition system and analysis method or not fully explored to be used in the coal mining industry. In this context, this research aimed to utilize the fundamental approach on understanding the MWD using seismic sensors by conducting experiments on a controlled laboratory environment. The samples for MWD were prepared from scratches for the prior knowledge of the drilled medium and to ease the interpretation. At the same time, the drill experiments were performed with the field scale drill machine at the laboratory. Both the mechanical and seismic sensors were utilized, and the recorded signals were analyzed using a Random Forest algorithm for classification and regression tasks, which was not reported in other literature on the application of MWD data analysis. An experimental workflow is shown in Figure 1.

## 2. Laboratory Experiment

Mechanical sensors to acquire drill signals, for example, displacement, rotary speed, torque, and load on bit, are commonly available on most drill machines. If not, they can be easily added onto the drill machine. These signals can be used to derive and interpret relevant parameters, such as, penetration rate (using feed rate, displacement and revolution per minute) [33,34]. The results from the mechanical sensors have been already discussed in [34]. A brief comparison of mechanical sensor data with the seismic sensor data is provided in this paper. The monitoring and response of these signals can offer valuable information on the geo-mechanical properties of the strata while drill bit penetrates the medium. For example, the pull-down force can provide strength and hardness of the rock. The pull-down force decreases in soft rock transitions and increases in harder-stronger transitions [35]. The intensity of vibration provides the strata type of different strengths. Torque provides variation between soft and hard rocks.

The experiments were conducted at the Rock Cutting Laboratory at CSIRO Queensland Centre of Advanced Technologies (QCAT) using a field scale drill rig. The detailed discussion and specifications of the drill machine are provided in [33].

For mechanical sensors, LabVIEW (Build 19) was used to record the raw data at every millisecond. Thus, from each drilling experiment, an enormous size of data file was produced. Similarly, seismic sensors, accelerometers, and geophones were also used for the experiments, and data were acquired at the sampling rate of 4000 Hz. Similar to mechanical sensors, the signals obtained from the seismic sensors also produced huge amounts of data. These data were manually inspected, trimmed, extracted and correlated with the time stamp from the mechanical sensors data.

### 2.1. Sample Preparation

**Figure 2:**
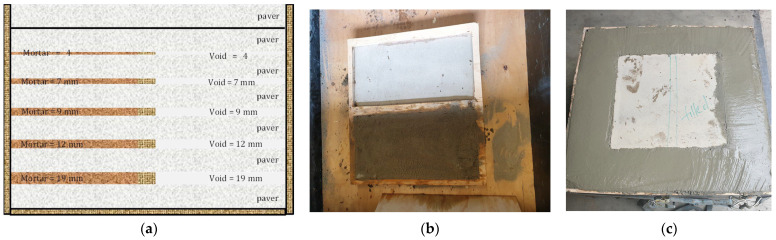
(**a**) Conceptual sample design separated by pavers. The pavers were 40 mm thick. The mortar and void regions were separated by a plywood. (**b**,**c**) Various stages of sample preparation at the laboratory.

The conceptual sample is shown in Figure 2a. The conceptual sample was prepared carefully with the precast stone pavers, and premixed mortar sourced from one of the landscape suppliers. It has to be noted here that the aim of the current experiment was to identify and quantify the thickness of the weak fractured interfaces, and the voids present in the sample. Hence, the mechanical properties of the pavers were irrelevant and were not measured. These precast pavers were relatively flat surface; hence, it was assumed that the thickness of the paver was almost constant throughout the paver and would maintain a constant interfacial gap between them. These pavers were stacked on top of each other by maintaining desired gaps in between them by using plywoods. The thickness of the plywoods that were used to maintain the desired gaps were as follows: 4 mm, 7 mm, 9 mm, 12 mm and 19 mm, respectively, from top to bottom. The pavers were 0.6 m by 0.04 m squares thick. On top, two pavers were stacked without any glue. The plywoods with the desired thickness were cut in the middle by leaving 10 mm on either side. The cut plywoods were stuck onto the paver using liquid nail to avoid any movement while stacking the pavers on top of each other. The mortar was a weak material just mixed with water and pasted in between the pavers, and the void area was maintained empty. After setting up the pavers and mortar, the assembly was pasted with silicon gel. This restricted any liquid from the concrete to penetrate the sample and avoid the conversion of mortar to the solid base. The various stages of sample preparation are illustrated in Figure 2. The weak mortar was selected to represent fractured and weak strata. This prepared sample was cased in a large timber outer casing framed by concrete structure of dimension 0.8 m square with 0.5 m height. The outer casing was needed to maintain the blocks firm during the drilling process.

### 2.2. Monitoring Configuration

**Figure 3:**
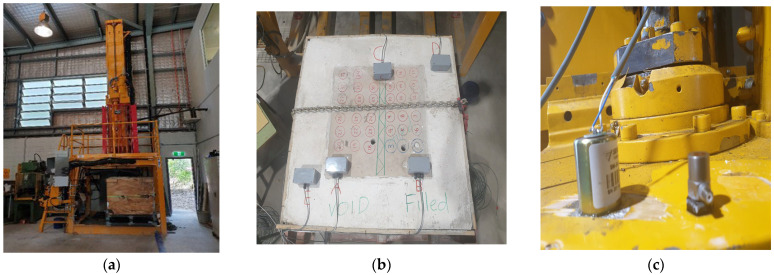
(**a**) Drill rig and experimental setup [33]. (**b**,**c**) Geophones and accelerometer sensor locations in the sample (**b**) and the drill head (**c**).

Normally, for the dominant frequence domain between 10 and 100 Hz, geophones are used for recording microseismic signals, whereas accelerometers are suitable for registering vibration signal with more broadband frequency ranging from 1 to 10,000 Hz. The geophones and accelerometers were installed at various locations on the sample and on the drill head to investigate the effect of the locations on the acquired signals, as demonstrated in Figure 3. Figure 3a also shows the experimental set up. The specifications of the geophones and accelerometers are listed in Table 1. The sensors were attached on the sample with gypsum and the drill head with the ethyl 2-cyanoacrylate, generally called as “super glue” and are available in the hardware shop. The sensors were connected to a National Instruments (NI) data acquisition system. A sample rate of 4000 Hz was configured for monitoring.

Drilling experiments were conducted with two different approaches:

- With a defined weight on bit and allowing for the feed rate to change;
- With a defined feed rate.

Two different sized drill bits (28 mm and 38 mm) were used to compare the influence of drill bit diameter on MWD. The selection of 28 mm and 38 mm drill bit sizes was referenced from the drilling machines that are used in the mining industry for roof drilling. The weight on bit varied from 1.5 kN to 94 kN. The RPM varied from 50 to 600. The feed rate varied from 8 mm/s to 32 mm/s.

## 3. Results

**Figure 4:**
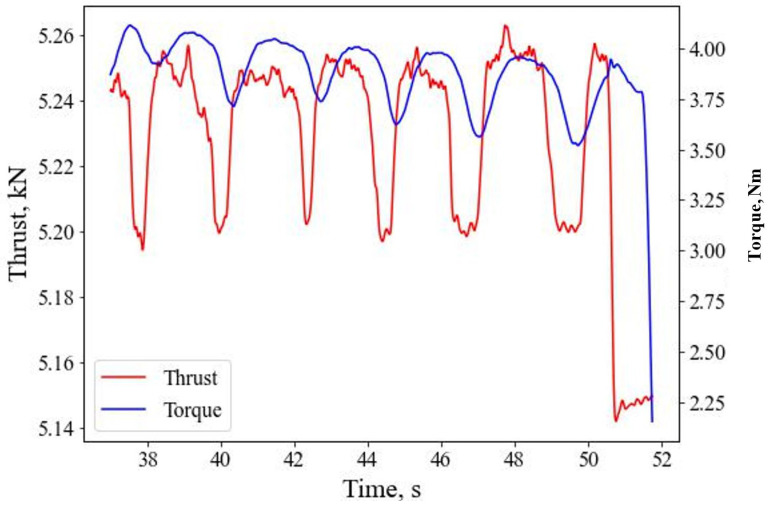
Thrust and torque signal with respect to drilling time.

Figure 4 shows the recorded signals for thrust and torque (mechanical sensor) at location “A” with reference to the drilling time. The figure shows that the mechanical sensor can identify the interfaces present in the samples. The larger the interface gaps, the larger the width of the lower amplitude of seismic signals. A detailed analysis of mechanical sensors data is provided in [34]. The pavers used in the sample are of the same thickness; hence, the time to each paver is approximately the same. As the relationship between torque and thrust is proportional to each other, the nature of the curves is similar. The slight time shift on the two curves could be due to the time delay in acquiring the signals between the sensors. However, in both curves, it can be noted that various penetrated layers can be easily identified, along with the interfacial distances between the pavers.

As a preliminary study on suitability of using geophones and accelerometers, each hole was drilled on either side of the sample representing filled and void areas. For ease of data interpretation, the installed sensors are identified as shown in Table 2.

**Figure 5:**
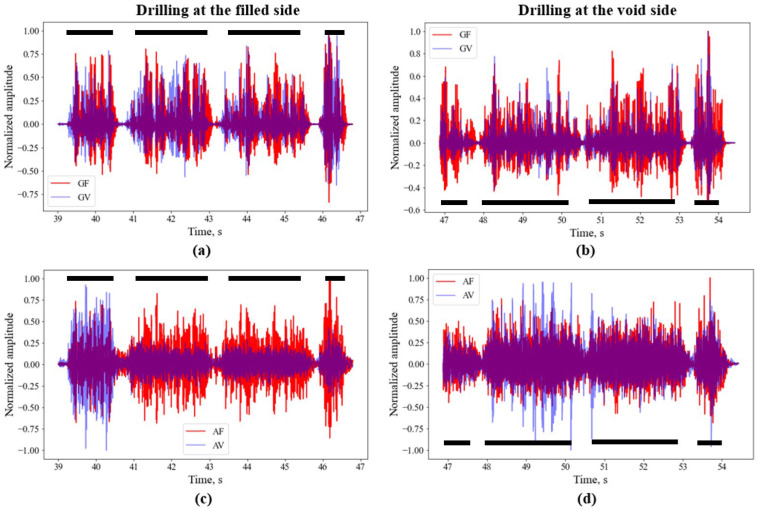
Signals from geophones and accelerometer sensors at two different locations. (**a**) Comparison of geophone signals between weak mortal filled side and void side at drill hole 1; (**b**) comparison of geophone signals between weak mortal filled side and void side at drill hole 2; (**c**) comparison of accelerometer signals between weak mortal filled side and void side at drill hole 1; and (**d**) comparison of accelerometer signals between weak mortal filled side and void side at drill hole 2. The black dashed lines show the approximate locations of the pavers.

Figure 5 shows the signals obtained from geophones and accelerometers at mortar filled (weak layers to represent fractured strata) and void sides of the sample during drilling. In the Y-axis, the normalized amplitude noted from geophones and accelerometers are plotted against the drilling time. The drill time is proportional to the drilling depth. Figure 5a,c shows the geophone signals and accelerometer signals, respectively, obtained from the filled “GF” and void “GV” sides while the drilling is conducted at the filled side. Similarly, Figure 5b,d show the geophone signals and accelerometer signals, respectively, obtained from the filled “GF” and void “GV” sides while the drilling is conducted at the void side. The figure shows that various drilled layers and the interfaces can be interpreted from the graphs. The gaps between the black solid lines show the location of the interfaces. The geophone signals represented by the top pictures have clearer demarcation between the layers compared to the accelerometer signals represented by the bottom pictures. There is not any significant difference between the mortar filled (weak layers to represent fractured strata) and void sides of the sample, suggesting that the geophones and accelerators cannot differentiate if the drilled medium is mortar filled (weak layers to represent fractured strata) or void.

**Figure 6:**
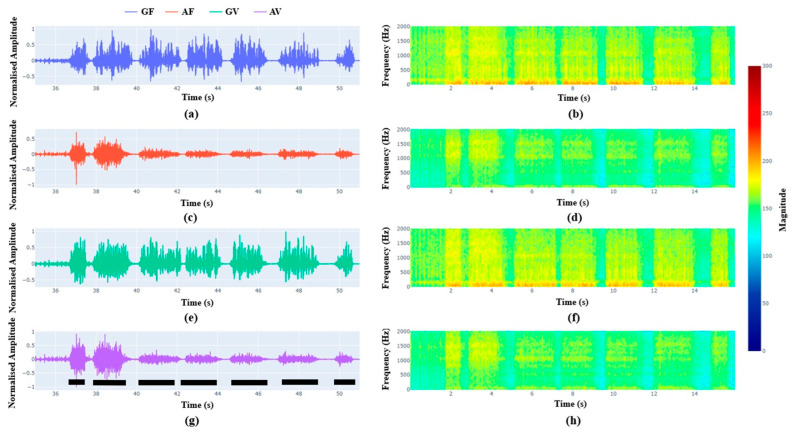
Comparison of geophone and accelerometer signals at different locations while drilling at the weak mortar side. (**a**,**b**) Geophone signal from weak mortar filled side; (**c**,**d**) accelerometer signal from weak mortar filled side; (**e**,**f**) geophone signal from void side; and (**g**,**h**) accelerometer signal from void side. The black dashed lines show the approximate locations of the pavers.

**Figure 7:**
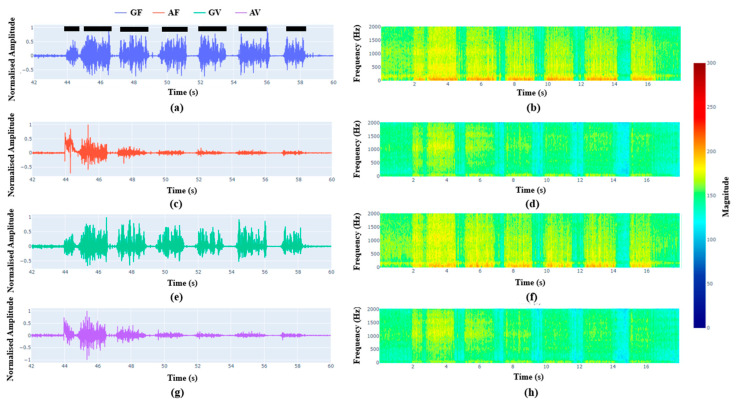
Comparison of geophone and accelerometer signals at different locations while drilling at the void side. (**a**,**b**) Geophone signal from weak mortar filled side; (**c**,**d**) accelerometer signal from weak mortar filled side; (**e**,**f**) geophone signal from void side; and (**g**,**h**) accelerometer signal from void side. The black dashed lines show the approximate locations of the pavers.

Figure 6 and Figure 7 show the vectorial representation of geophones and accelerometers signals from the filled and void sides. Compared to Figure 5, these figures show clear demarcation between pavers and interfaces. The larger the interfaces, the larger the width of the low-amplitude signals. From the pictures, it is further affirmed that the geophones and accelerators cannot differentiate between mortar filled (weak layers to represent fractured strata) or void interfaces. The seismic sensors showed the capacity to identify the fractures or weak interfaces up to 4 mm thin in the tested condition, particularly geophone signals are clearer compared to the accelerometer signals. Smaller interfaces had a smaller span of low amplitude and vice versa. Within the tested domain, geophones seemed to be better than accelerometers in identifying interfaces. The geophones show the distinct demarcation from 4 mm to 19 mm. However, accelerometers seem to show distinct demarcation only up to 9 mm thick void and interfaces. For the interfaces of thickness less than 9 mm, accelerometers show a lot of noise at the interfaces. This could be due to the nature of accelerometers which may be more sensitive to vibration and prone to more noise signals. Hence, signals recorded by geophones are more suitable for interface identification than that of accelerometers.

**Figure 8:**
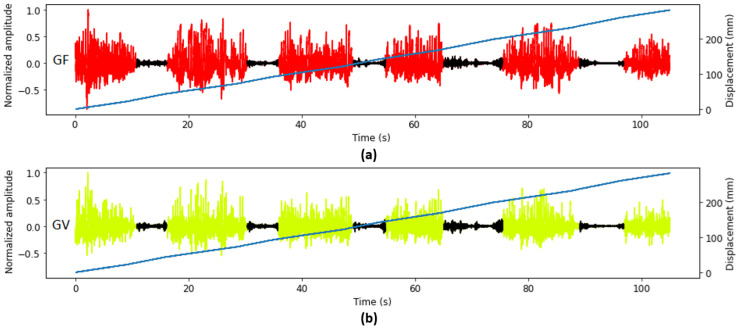
An example of recorded seismic (geophone) signals and displacement: (**a**) geophone signal from the weak mortar filled side, and (**b**) geophone signal from the void side. The black and blue lines indicate the hand-labeled seismic signals registered during the voids being drilled and displacement of the drill, respectively.

To demonstrate that geophone signals can be used for automatic void identification, the seismic signal (geophone signal) was manually labeled as rock and void with respect to the variations in signal amplitude (Figure 8). The black portion in the graphs show the hand-labeled location to represent the presence of interfaces. The blue line shows the displacement of the drill rod during drilling. The thickness of each paver and void was obtained based on the displacement of the drill corresponding to the labeled signal sections. It was found that the thicknesses of the pavers and voids were not the same as designed. This could be due to the self-weight of the pavers or uneven thickness of the pavers which caused non-uniform interfacial thickness between the pavers. The manually labeled thicknesses were used as a benchmark to compare with the predicted thicknesses of the pavers and voids.

Random Forest [36] is a widely used machine learning algorithm for both classification and regression tasks. It is an ensemble of decision trees created with randomly and uncorrelated features [36]. Each decision tree is trained on a different subset of the data, with a different subset of the features randomly selected for each tree. This helps reduce overfitting and increase the model’s generalization performance. In the case of classification tasks, the final prediction of the Random Forest algorithm is based on the majority vote of the individual decision trees. This method was applied in this experiment to identify if the recorded seismic signal is associated with the drilling through a paver or a void. By correlating the displacement with the classified seismic signal, the thickness of the pavers and voids were predicted.

**Figure 9:**
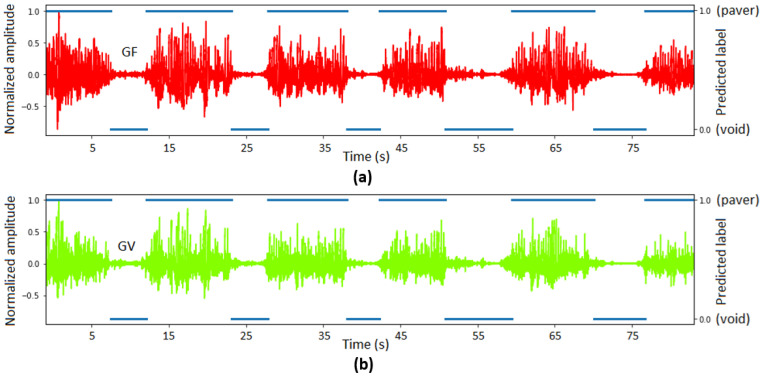
Seismic signal recorded by geophones: (**a**) geophone signal from the weak mortar-filled side, and (**b**) geophone signal from the void side, while drilling another hole at the void side and the associated labels were predicted by the Random Forest classifier. The blue line indicates the predicted label.

**Figure 10:**
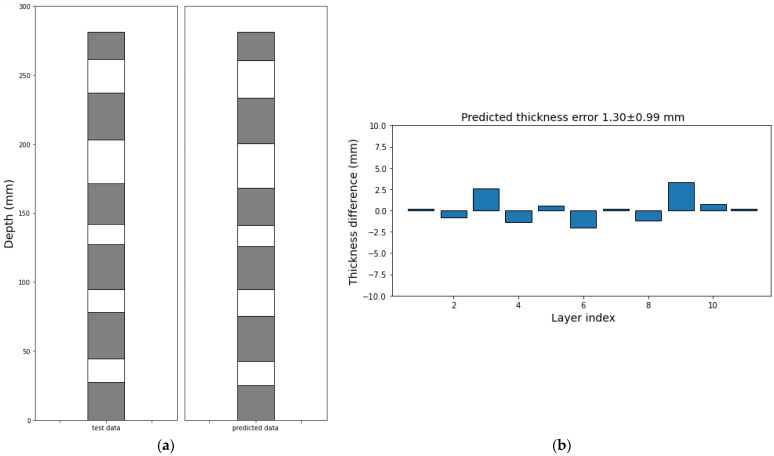
(**a**) Benchmark and predicted layers; (**b**) difference between the benchmark and predicted thickness of each layer.

To train a Random Forest model to predict the thicknesses of pavers and voids, the seismic signal recorded from 1 drill at the void side was hand labelled as 1 and 0, indicating seismic signal associated with drilling through pavers and voids, respectively. It was split to 80% and 20% to train and test the Random Forest classifier. The test achieved excellent performance with 97% accuracy. The trained model was applied to the seismic signal recorded during another drill. The predicted results are shown in Figure 9. The thickness of all identified layers was compared with the benchmark thicknesses, and the differences of thicknesses are shown in Figure 10. The results show that the average error of predicted thickness of each layer is 1.3 mm, suggesting that seismic signals can accurately identify paver and void with uncertainty less than 3 mm during the lab experiment. As a future work, this observation is planned to be tested in the laboratory using a high-resolution borehole inspection camera that can detect millimeter range distance in a lo-light region.

The discrepancy in interface thickness during sample preparation and automatic detection could be due to the designed interface thickness changed during drilling.

**Figure 11:**
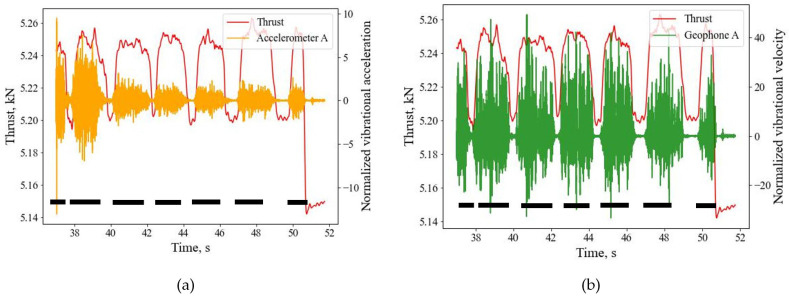
Signals detected from the (**a**) accelerometer; (**b**) geophone, and comparison with the thrust and torque sensors at location A. The black dashed lines show the approximate locations of the pavers.

One of the purposes of using geophones and accelerometers was to find out if these seismic sensors could be efficiently used to complement force and torque sensors in identifying characteristics of the drilled medium. Figure 11 shows the characteristics of thrust, geophone and accelerators from the representative drillings. As thrust and torque are linearly related, only the geophones and accelerometers signals are compared with thrust. The illustrations are referenced with the geophone and accelerometer located at location “A” (Table 2). As noted in the above figures, these graphs also show that the geophone and accelerometer can identify the interfaces and provide additional assurance to the interfaces detected by thrust and torque sensors, as interfaces larger than the experimented 4 mm width can easily be detected, geophones are mainly good at detecting thinner interfaces. Compared to seismic sensors, the boundaries of interfacial widths are bit fuzzy in signals obtained from the thrust sensors, particularly when the widths are smaller. However, the boundaries of interfacial widths are relatively clearer with the geophone signals, and can be related to the size of the interfaces present in the sample. Both types of sensors, thrust, and geophone and accelerometer sensors, could not differentiate between mortar-filled (weak layers to represent fractured strata) and void interfaces.

**Figure 12:**
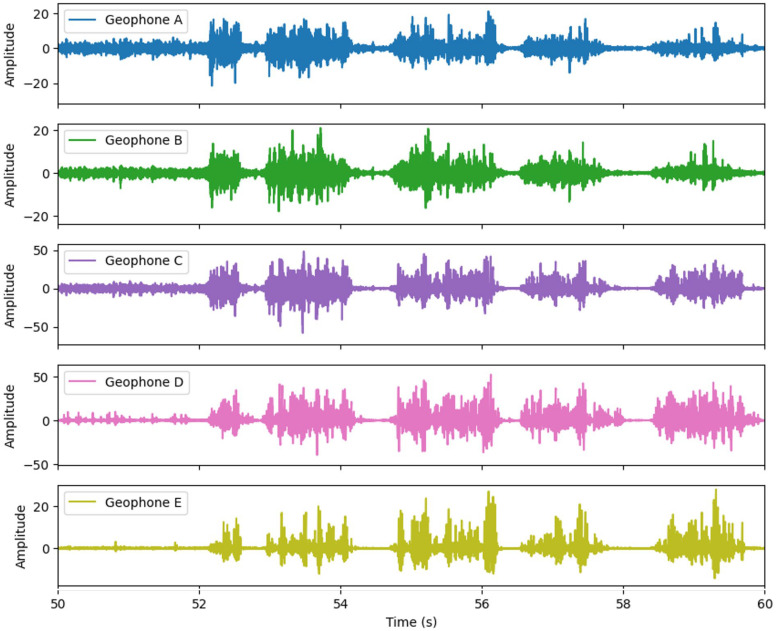
Geophone signals detected at different locations.

Figure 12 shows the comparison of signals from geophones installed at various locations during drilling at the same location. The main purpose of this comparison was to demonstrate that all the geophones can detect signals that can be characterized to identify interfaces. The results shown in the figure demonstrate that all the geophones can detect the signals. The hole was in the filled side, and the location of sensors is shown in Figure 3 and Table 2. Depending on the location of the sensors, the frequency and amplitude seemed to differ. This suggests that the location of the sensors is not an issue, but the intensity of the signals could vary. Therefore, in the current experiment, within the tested condition in the laboratory, the location of the seismic sensors has a lesser impact on interface identification.

**Figure 13:**
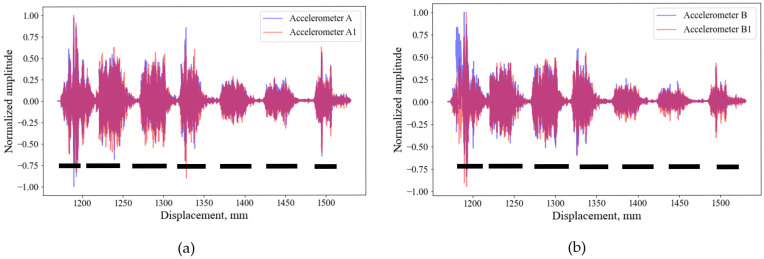
Comparison of two types of accelerometers at mortar-filled side (**a**) and void side (**b**). The black dashed lines show the approximate locations of the pavers.

Two different types of accelerometers (Table 1) were also tested to compare if the sensitivity of the tested accelerometers had any impact on the signal acquisitions (Figure 13). The figures demonstrate that both types of sensors can acquire similar data.

In the field work, it would be practical to attach seismic sensors on drill machine, particularly on the drill head. Therefore, some additional experiments were conducted by placing geophones and accelerometers on the drill head of the machine (location F, Figure 3 and Table 2). However, from experiments it has been noted that the signals obtained from the geophone and accelerometer attached on the drill head are not informative compared to those attached on the sample. This could be due to the intensity of vibration in the drill head and signals getting attenuated.

**Figure 14:**
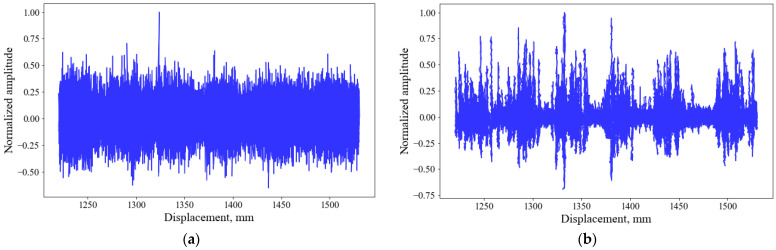
Seismic measurements from the drill head: (**a**) the accelerometer signal, and (**b**) the geophone signal recorded at the drill head.

Figure 14 shows the signals obtained on the drill head. From the graphs, it can be noted that the accelerometers do not sense the required signals to differentiate the drilled pavers and interfaces. However, geophones are relatively better in differentiating the pavers and interfaces compared to the accelerometers.

## 4. Discussion

From the experiments (Figure 4), it has been observed that the mechanical sensors, for example, thrust, torque, and rotary speed, can identify the interfaces present in the samples, larger the interfacial gaps, clearer the identification. Such sensors are also fitted in with most of the current drill machines that are used in the mining industry. The mechanical sensors also have the capacity to identify various layers that are present in the drilling medium. From a practical point of view, it would be appropriate to collect and analyze the data to characterize the drill medium. However, due to some reasons, such as a lack of confidence in the current data acquisition system and analysis method, the mechanical data were not fully utilized. In the context of interface detection during strata drilling, the main limitations of the mechanical sensors data can be identified as follows:

- Fuzzy demarcation between the interfacial boundaries;
- Inability to clearly characterize smaller interfaces.

To overcome these limitations, two different types of seismic sensors were tested during the laboratory drilling experiments. Compared to the mechanical sensors, the results shown in Figure 5, Figure 6 and Figure 7 demonstrate that the seismic sensors can provide clearer demarcation between the interfaces. Up to the tested thickness of 4 mm, both the seismic sensors, geophones and accelerometers show ability to identify the interfaces. Comparison between geophones and accelerometers shows that the former is clearer compared to the latter in interface identification. The accelerometers are more sensitive to vibrations and may get attenuated by the in situ or ground vibration during drilling. The identification of interface is mainly based on the span of the low frequency regions in the signal.

From all the experiments, it was noted that none of the tested sensors showed any capacity to differentiate the mortar filled (weak layers to represent fractured strata) and void sides of the sample. The seismic sensors data have a high noise to signal ratio, which possess a challenge to adopt a suitable data analysis approach.

The current study focuses on laboratory scale experiments with lot of simplifications and assumptions. For example, the assumptions of flat interfaces between the strata and uniform and leveled strata thickness. These simplifications and assumptions were needed to investigate the problem from the fundamental approach under controlled laboratory environment.

Compared to the laboratory experiments, the field conditions are complex; hence, the current approach of data analysis has to be refined before implementing in the field measurements. The synthetic concrete samples used in this study are relatively homogeneous compared to the actual rock samples that are encountered in field. Hence, the results and interpretation discussion presented in this paper may have to be further refined to analyze the MWD signals obtained from real rock drilling in the field.

In this paper, the Random Forest machine learning algorithm has been used. There are a number of other tools which may be suited for MWD signal processing application and may provide better interpretation. Hence, the current work is still progressing to identify the suitable data processing method.

The next step of the research is the application of the above-discussed methodology in the field and refine the data analysis methodology to gain confidence in interpreting interfaces present in the stratified geology.

## 5. Conclusions

One of the major challenges in measurement while drilling (MWD) research is accurately detecting voids, fractures, and thin layers within the strata. To address the identification of thin voids or weak zones as the drill bit penetrates the medium, a novel experimental setup was developed incorporating seismic sensors.

Alongside conventional mechanical sensors, such as torque and thrust, non-mechanical sensors, including geophones and accelerometers, were employed to classify drilled layers. Laboratory-synthesized blocks were constructed in stratified layers, with voids and interfaces introduced to simulate lithological units.

Raw drill signals collected during these experiments were processed, analyzed, and interpreted to determine whether they could provide characterization of the drilled medium. The results indicate that drill signals can characterize the penetrated material, including the interfaces between synthetic rock layers.

Distinct boundaries between sandstone layers and interfaces were identified using geophone and accelerometer data. For interfaces up to 4 mm thick, both seismic sensors demonstrated the ability to detect fractures or weak zones, although accelerometer signals were less clear than those from geophones. Sensors mounted directly on the sample were more sensitive than those attached to the drill head, likely due to vibration-induced signal attenuation at the drill head. Both the seismic sensors were unable to differentiate between weak fractured zones (filled areas) and actual voids.

## Figures and Tables

**Table 1:** Key specifications for sensors.

	Accelerometer A	Accelerometer B	Geophone
Frequency	1.0 to 10,000 Hz (frequency range)	0.5 to 15,000 Hz (frequency range)	10 Hz (natural frequency)
Sensitivity	1.0 mV/(m/s^2^)	10.2 mV/(m/s^2^)	28.8 V/m/s
Size	11.4 mm (Length) × 6.4 mm (Width) × 3.6 mm (Height)	11.2 mm (Length) × 11.2 mm (Width) × 15.7 mm (Height)	32 mm (Height) × 25.4 mm (Diameter)
Measurement Range	±4900 (m/s^2^)	±490 (m/s^2^)	
Weight	0.6 g	5.8 g	74 g

**Table 2:** Preliminary sensor location identification. Locations A, B, C, D and E are on the drill sample and are shown in Figure 3. Location F is on the drill head.

Name	Location
GF	geophone filled side
AF	accelerometer filled side
GV	geophone void side
AV	accelerometer void side
GI	geophone at the filled and void interface
GD	geophone on the drill head
AD	accelerometer on the drill head

## Data Availability

Data are contained within the article.
